# MADET: a Manually Curated Knowledge Base for Microbiomic Effects on Efficacy and Toxicity of Anticancer Treatments

**DOI:** 10.1128/spectrum.02116-22

**Published:** 2022-10-18

**Authors:** Jie Zhang, Xiqian Chen, Jiaxin Zou, Chen Li, Wanying Kang, Yang Guo, Sheng Liu, Wenjing Zhao, Xiangyu Mou, Jiayuan Huang, Jia Ke

**Affiliations:** a Center for Infection and Immunity Studies, School of Medicine, Shenzhen Campus of Sun Yat-Sen University, Shenzhen, Guangdong, China; b Independent Researcher, Beijing, China; c Department of Colorectal Surgery, The Sixth Affiliated Hospital, Sun Yat-Sen University, Guangzhou, Guangdong, China; d Guangdong Provincial Key Laboratory of Colorectal and Pelvic Floor Diseases, The Sixth Affiliated Hospital, Sun Yat-Sen University, Guangzhou, Guangdong, China; e Monash Biomedicine Discovery Institute and Department of Biochemistry and Molecular Biology, Monash University, Melbourne, Victoria, Australia; Shenzhen Bay Laboratory

**Keywords:** database, microbiota, anticancer treatment, efficacy, toxicity

## Abstract

A plethora of studies have reported the associations between microbiota and multiple diseases, leading to the development of at least four databases to demonstrate microbiota-disease associations, i.e., gutMDisorder, mBodyMap, Gmrepo, and Amadis. Moreover, gut microbiota mediates drug efficacy and toxicity, whereas a comprehensive database to elucidate the microbiota-drug associations is lacking. Here, we report an open-access knowledge base, MADET (Microbiomics of Anticancer Drug Efficacy and Toxicity), which harbors 483 manually annotated microbiota-drug associations from 26 studies. MADET provides user-friendly functions allowing users to freely browse, search, and download data conveniently from the database. Users can customize their search filters in MADET using different types of keywords, including bacterial name (e.g., Akkermansia muciniphila), anticancer treatment (e.g., anti-PD-1 therapy), and cancer type (e.g., lung cancer) with different types of experimental evidence of microbiota-drug association and causation. We have also enabled user submission to further enrich the data documented in MADET. The MADET database is freely available at https://www.madet.info. We anticipate that MADET will serve as a useful resource for a better understanding of microbiota-drug associations and facilitate the future development of novel biomarkers and live biotherapeutic products for anticancer therapies.

**IMPORTANCE** Human microbiota plays an important role in mediating drug efficacy and toxicity in anticancer treatment. In this work, we developed a comprehensive online database, which documents over 480 microbiota-drug associations manually curated from 26 research articles. Users can conveniently browse, search, and download the data from the database. Search filters can be customized using different types of keywords, including bacterial name (e.g., Akkermansia muciniphila), anticancer treatment (e.g., anti-PD-1 therapy), and cancer type (e.g., lung cancer), with different types of experimental evidence of microbiota-drug association. We anticipate that this database will serve as a convenient platform for facilitating research on microbiota-drug associations, including the development of novel biomarkers for predicting drug outcomes as well as novel live biotherapeutic products for improving the outcomes of anticancer drugs.

## INTRODUCTION

Gut microbiota plays an important role in carcinogenesis and cancer treatment outcomes (1, 2). Bacteria, including Helicobacter pylori (3), Fusobacterium nucleatum (4), Peptostreptococcus anaerobius (5), enterotoxigenic Bacteroides fragilis (6), polyketide synthase-positive (*pks*^+^) Escherichia coli (7), and Campylobacter jejuni (8), have been reported to contribute to carcinogenesis and tumor development via releasing toxins and activating procarcinogenic signaling pathways (1, 2, 4). On the other hand, microbes, including Lactobacillus reuteri (9), Lactobacillus gallinarum (10), and Streptococcus thermophilus (11) are reported to suppress tumorigenesis and cancer progression via multiple pathways, such as inhibiting the metabolism of tumor proliferation (11). To date, several databases have been constructed to document these positive or negative associations between gut microbiota and tumor development, such as mBodyMap (12), gutMDisorder (13), GMrepo (14), and Amadis (15). However, the important effects of microbiota on the efficacy and toxicity of anticancer drugs have not been systematically documented to date.

Gut microbiota modulates the efficacy and toxicity of anticancer drugs through multiple mechanisms, including immunomodulation, metabolism, and enzymatic degradation (16). Notably, the efficacy of immunotherapies (e.g., anti-PD-1 therapy) in cancer patients is positively associated with the abundance of some specific bacterial species (e.g., Akkermansia muciniphila [17], Bacteroides fragilis and Bifidobacterium longum [18]). These commensal gut microbes may reinforce the efficacy of immune checkpoint inhibitors (ICIs) via regulation of the host immune responses (such as increased level of CD4^+^ T cells and/or CD8^+^ T cells in the tumor microenvironment) (17, 19). Furthermore, fecal microbiota transplantation has been demonstrated to promote efficacy of anti-PD-1 therapy in immunotherapy-refractory melanoma patients (20, 21). Several live biotherapeutic products are under development for their synergistic anticancer effects with immunotherapies, and the clinical trials based on these bacterial products are ongoing, including CBM588 (a Clostridium butyricum strain) for treating advanced kidney cancer (22). Moreover, the toxicities of anticancer treatments are impacted by gut microbiota. For example, chemotherapeutic agent irinotecan may be metabolized by gut bacterial β-glucuronidase (which can be found in four major bacterial phyla: *Bacteroidetes, Firmicutes, Verrucomicrobia*, and *Proteobacteria*) into its toxic form SN-38, and therefore may inflict toxicity in the gastrointestinal tract, such as epithelial damage and diarrhea (23). Another example is that immune-related adverse effect (irAE) of ICI therapy, most commonly colitis, has been reported to be associated with the abundance of the *Bacteroidetes* phylum in the human gut (24). In a nutshell, microbiota could be employed as a potential biomarker for predicting treatment outcomes and an interventional target for improving the effectiveness of cancer therapies. We therefore argue that with the accumulating evidence of the important roles of microbiota in anticancer treatments, including chemotherapeutic agents, radiotherapy, and immunotherapies, it is urgent to develop an open-access and user-friendly knowledge base to allow for documenting, retrieving, and sharing experimental data of these microbiotas and their effects on the efficacy and toxicity of anticancer drugs.

**FIG 1:**
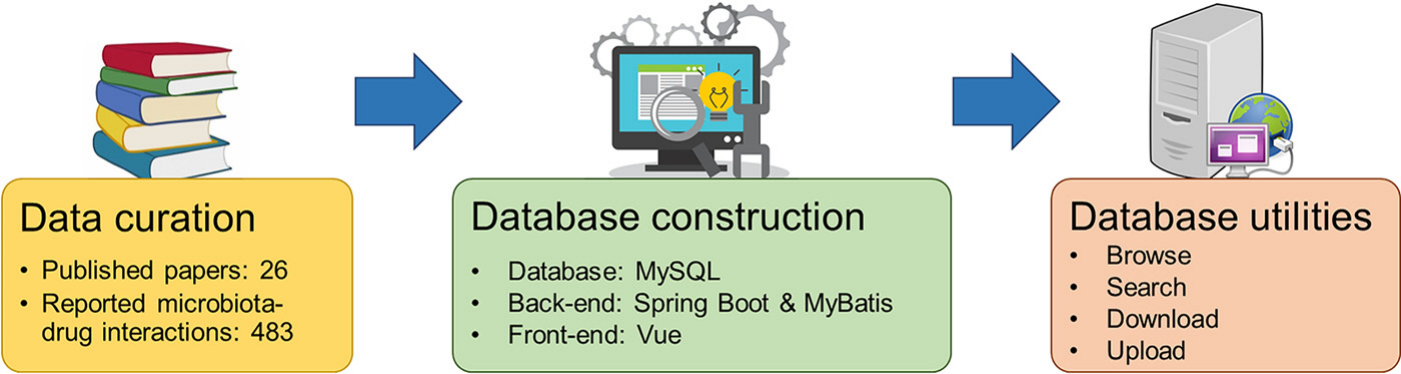
The framework of MADET, including data curation, database construction and utilities.

To this end, in this study we implement a knowledgebase, termed MADET (Microbiomics of Anticancer Drug Efficacy and Toxicity; freely available at https://www.madet.info), to provide the most up-to-date manual curation of reports on the interplays between microbiota and anticancer drugs for the researchers in microbiology, pharmacology and other related areas (Fig. 1). To the best of our knowledge, MADET is the first database providing experimental evidence of associations between microbiota and anticancer drugs with user-friendly functionalities, including data download and flexible keyword search with bacterial name, treatment, or cancer type. We anticipate MADET will serve as a steppingstone for the development of novel microbiomic biomarkers for predicting the outcome of anticancer drugs and live biotherapeutic products for improving the outcome of anticancer drugs.

## RESULTS

### MADET utilities.

**FIG 2:**
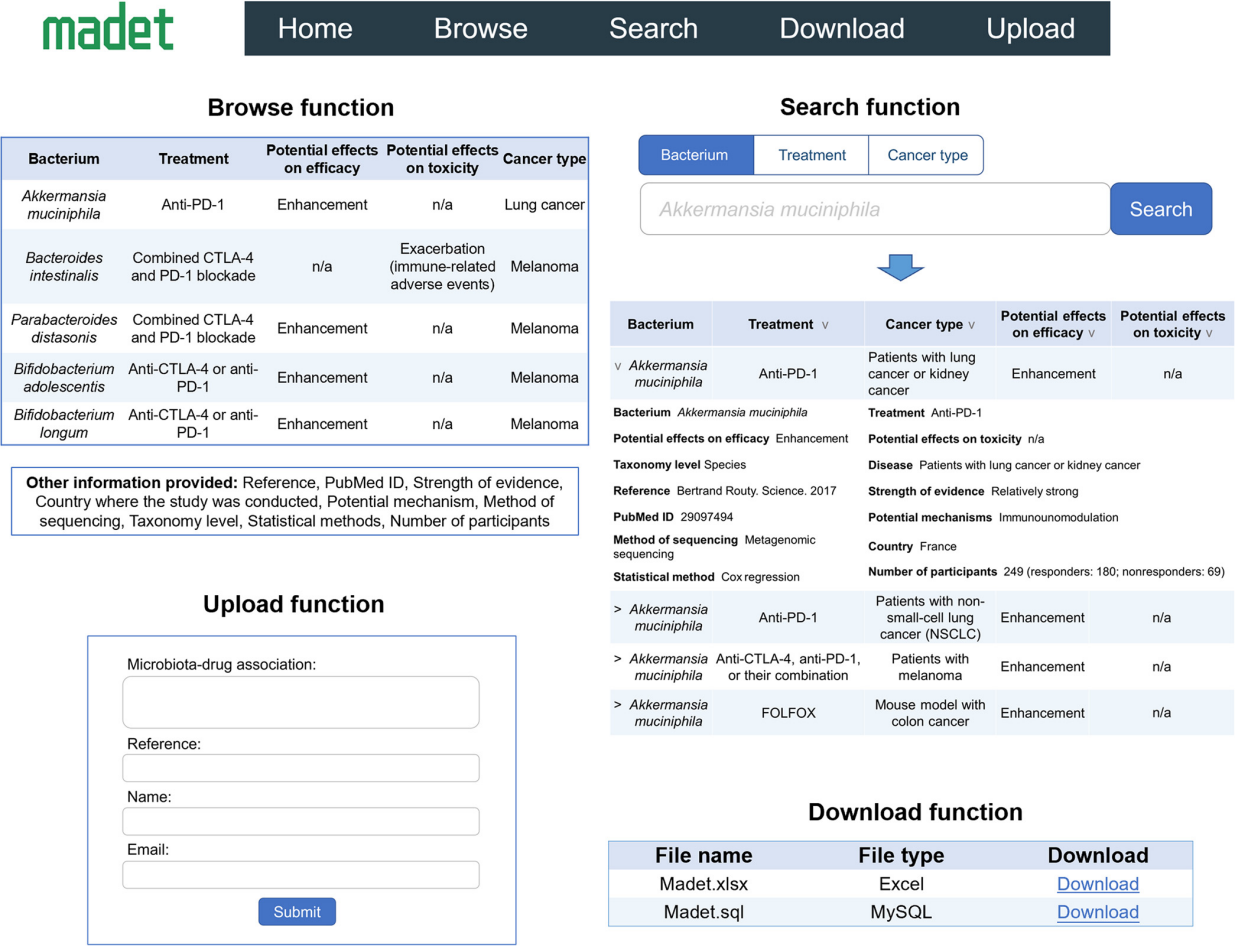
Major functions provided in MADET, including browsing, searching, downloading, and uploading.

MADET provides four major functionalities, including database browsing, searching, downloading, and user submission with a user-friendly interface (Fig. 2). The home page contains two sections. The top section provides a brief introduction to the MADET database and the generic statistical information, such as treatment and cancer type, of the current MADET version using interactive pie charts. Users can click on each pie chart to be redirected to corresponding result pages. For example, if “China” from the pie chart is clicked, MADET will show filtered search results that contain studies conducted in China. Four related links (i.e., mBodyMap, gutMDisorder, GMrepo, and Amadis) are displayed at the bottom of the home page for redirecting users to other referred databases directly. The “Browse” page enables users to view all data available on MADET and allows users to set customized filters to shortlist the results. Each MADET entry contains 16 types of information, including “Bacterium,” “Treatment,” “Potential effects on efficacy,” “Potential effects on toxicity,” “Cancer type,” “Strength of evidence,” “Taxonomy level,” “Method of sequencing,” “Potential mechanisms,” “Country,” “PubMed ID,” “Number of participants,” “Statistical method,” “Sampling site,” and “Other information.” Critical information is shown by default, and complete information is shown upon clicking on the drop-down button. Several column headers are provided with an arrow, clicking on which will show a drop-down menu of subcategories for users to narrow down the results. The filter will auto-apply when a new attribute is selected, and the table will auto-refresh to display the filtered results. Multiple subcategories from different attributes can be selected at the time for customized filters. If the number of entries exceeds the limit on one page, users can click the pages button at the bottom of the table to scroll through the data. Searching microbiota-drug interactions of interest is straightforward. Three types of searching are available on the “Search” page, including “Bacterium,” “Treatment,” and “Cancer type”. After obtaining the desired keywords provided by the user, the search engines will only search the database entries with the provided keywords. We have also provided default keywords for each search type. Users can simply click the “Search” button to see the results extracted using the default keywords. The search results are displayed as a table similar to the table on the “Browse” page. A drop-down menu is provided from “Treatment” and “Cancer type,” respectively, to narrow down the search results of interest. Users can click the “Advanced” button below the search box to enter the advanced search function, which supports searching by logical operation for a more precise search. MADET allows users to download its data for further analysis. Users can download the sql (MySQL) or xlsx format of the data collected by MADET through the “Download page.” To further enrich the data documented in MADET, we have enabled users to submit their own research data via our submission function on the “Upload” page. We request the users to describe the information that should be covered in our database in detail, including treatment, bacterium, and cancer type. The submitted data will be manually reviewed by the MADET team prior to data publishing in the database. The users will be contacted if more clarification is needed via their name and email address provided during data submission. We will confirm with the users once their data have been accepted for publishing in the MADET database.

### Case study.

Some interesting findings involving microbiota-drug associations can be discovered using our MADET database. First, certain bacterial species may exert opposite effects on modulating the efficacy of different drugs. For instance, by searching MADET with the keyword “Fusobacterium nucleatum,” we are able to see that the bacterial species F. nucleatum is negatively associated with the decreased anticancer effects of 5-FU chemotherapy for colorectal cancer (25) as well as positively related to the promoted efficacy of PD-L1 blockade on the other hand (19). This suggests that a comprehensive evaluation may be needed prior to the administration of next-generation probiotics targeting microbiota-drug association.

Second, even for the same anticancer drug, we found that same bacterial species showed opposite potential effects against different cancer type or across different countries. For example, searching “Roseburia intestinalis” in MADET showed this species is positively associated with the efficacy of immune checkpoint inhibitor (ICI) therapy in patients with lung cancer in France (26), whereas *R. intestinalis* was reported to be negatively associated with the efficacy of ICI therapies in melanoma patients in the United States (18). Similar results on Parabacteroides distasonis, Lactobacillus vaginalis, and Eubacterium hallii can be unveiled through searches in MADET with relevant keywords. These findings highlight that factors such as cancer types, geographic locations, and analytical methods may affect the conclusion of the microbiomic effect on anticancer drugs. Moreover, the functional differences may be strain dependent, i.e., heterogeneous bacterial strains within the same species may have different functions on drug outcome (27). Thus, MADET provides a useful platform to summarize and analyze the precise mechanism of the observed microbiota-drug associations demonstrated in the current literature.

## DISCUSSION

With the rapid expansion of our knowledge of gut microbiota during the past decade, multiple bacterial species have been identified as promising candidates for biomarkers of drug susceptibility and live biotherapeutic products for improving drug outcomes. By manually curating 483 associations between microbiota and anticancer drugs with a variety of factors including bacterial species, treatment, cancer type, and strength of evidence, the MADET database provides the first comprehensive knowledge base of the associations between microbiota and anticancer therapeutic outcomes. Users can utilize MADET to examine the up-to-date microbiota-drug associations of their bacterium/drug of interest, to advance the generation of novel research hypotheses. The curated data in MADET show that our current understanding on pharmacomicrobiomics is mostly based on observational studies, highlighting that future mechanistic and causality research is needed. MADET will keep evolving with the new data from novel studies on microbiota-drug associations. We anticipate that MADET will serve as a convenient platform for facilitating pharmacomicrobiomics research, including developing novel biomarkers for predicting drug outcomes as well as novel live biotherapeutic products for improving the outcomes of anticancer drugs.

## MATERIALS AND METHODS

### Data collection.

**FIG 3:**
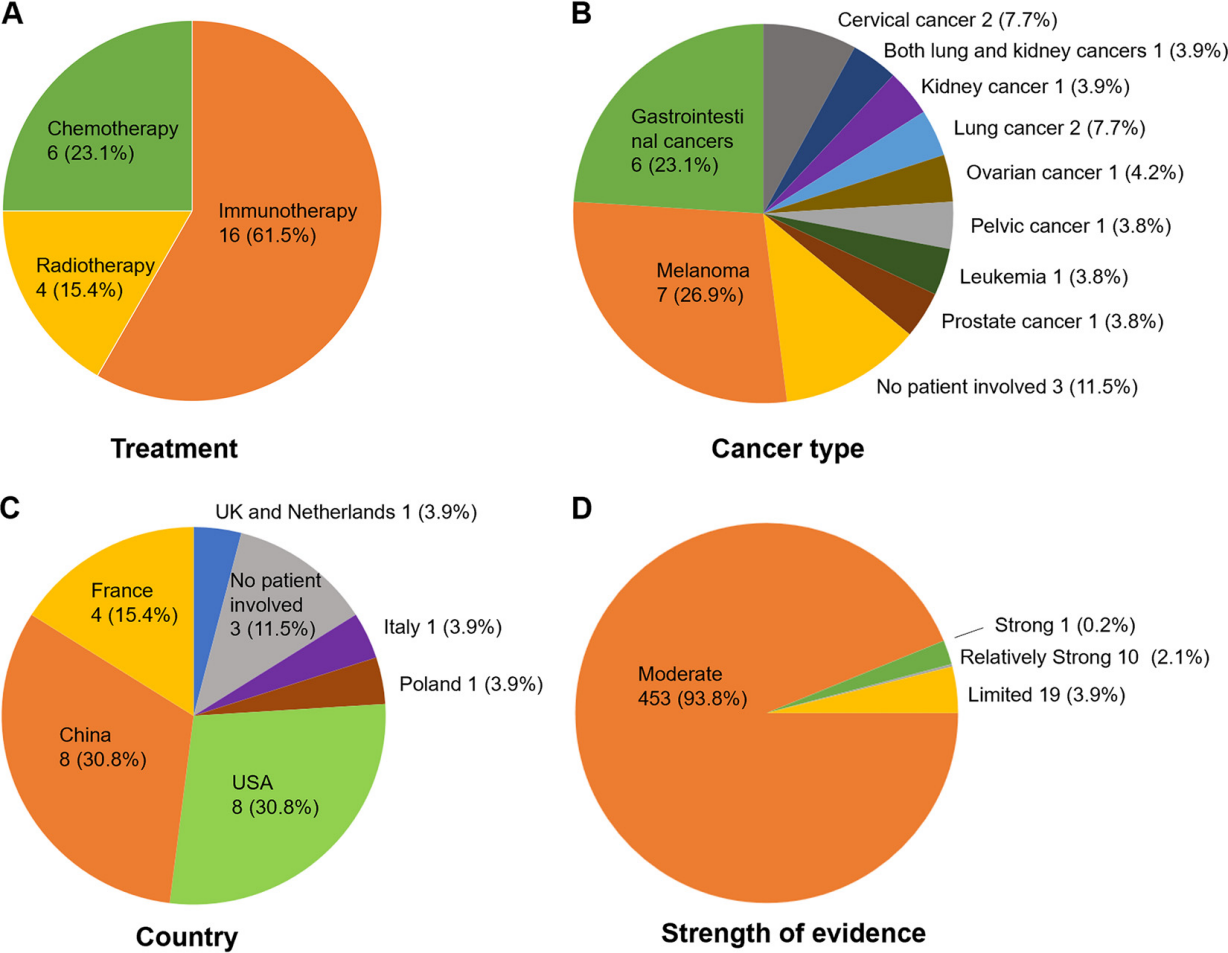
Statistics of curated studies for microbiota-drug associations. The collected studies have been categorized by (A) treatment approaches, (B) cancer types (one study recruited both kidney cancer patients and lung cancer patients), (C) countries where the study was conducted, and (D) the strength of evidence.

To collect the state-of-the-art scientific reports on pharmacomicrobiomics of anticancer drugs, we searched the literature using the keywords “microbiome” and “cancer treatment” (e.g., 5-FU, anti-PD-1 or radiotherapy, etc.) on PubMed and extracted 26 published papers involving how the composition of microbiota is associated with the efficacy and toxicity of anticancer drugs. Sixteen of the 26 papers focused on the associations between microbiota and ICIs, and six papers examined the effects on chemotherapeutic agents and their combinations (Fig. 3A). We further included four publications on cancer radiotherapy studies, given the fact that radiotherapy has been widely applied to cancer treatment. In total, 483 associations between microbiota and drug outcome have been manually identified and recorded in MADET.

We also analyzed our collected studies based on the cancer type and the country where the research was conducted (Fig. 3B and C). Melanoma and gastrointestinal cancers attracted major attention from anti-cancer pharmacomicrobiomics researchers, which comprise seven and six papers, respectively. We further set up several criteria in terms of experimental types for categorizing the collected 483 microbiota-drug associations: “Limited” (only with animal data); “Moderate” (clinical observation of association); “Relatively strong” (clinical observation of association and experimental validation of causation using animal model); and “Strong” (clinical observation of association and validated of causation by clinical trial). Among those, the majority (453 associations) have a “Moderate” strength of evidence (93.8%) (Fig. 3D). Only one association was labeled as “Strong”: a Clostridium butyricum strain CBM 588 increases the effect of immunotherapy (nivolumab and ipilimumab) in treating patients with kidney cancer, which was validated by a Phase 1 clinical trial (22). These statistics suggest that our current knowledge of pharmacomicrobiomics is mostly from observational association studies, whereas more in-depth research is needed to explore the causality and mechanism behind the interactions between microbes and drugs.

### MADET construction.

MADET was developed and implemented utilizing widely applied open-source packages including Vue (front-end), Spring Boot and MyBaits (back-end), and MySQL (relational database design), to provide reliable delivery of a complex scalable web application (Fig. 1). The modern and separate front-end/back-end architecture of MADET allows collaboration between developers with different specializations and simplifies the upgrading process for future improvement. The main proxy uses a high-performance Nginx server (https://www.nginx.com) to communicate between the front-end and the back-end programs. The front-end of MADET was constructed with a commercial-used open-source Vue.js framework (https://vuejs.org) and Element UI Toolkit (https://element.eleme.io) for the design of a professional user interface experience design. The back-end of MADET has been designed to be a microservice architecture software, which is facilitated by Java Spring Boot (https://spring.io/projects/spring-boot). The microservice architecture implemented three independent functions modules (services), i.e., the search, browse, and upload services. This highly modular architecture allows MADET to provide software as a service (SaaS) and the possibility of turning MADET into a mobile app and adding new services in the future. The actual data stored in MySQL (sql) can be easily converted to other data formats such as xlsx (Microsoft Excel) and csv (comma separated values). The MADET website resides on Tencent Cloud to leverage the burden of various website configurations and security measures while preserving full control of the web application and data integrity to MADET developers.

### Data availability.

The MADET database is freely available at https://www.madet.info. Any code or other data related this work will be available upon reasonable request.
